# Acute Pulmonary Edema After Hyperbaric Oxygen Treatment: A Case Report Written With ChatGPT Assistance

**DOI:** 10.7759/cureus.34752

**Published:** 2023-02-07

**Authors:** Haris M Akhter, Jeffrey S Cooper

**Affiliations:** 1 Hyperbaric Medicine, University of Nebraska Medical Center, Omaha, USA

**Keywords:** diabetes, heart failure with reduced ejection fraction, acute pulmonary edema, chatgpt, hyperbaric oxygen therapy (hbot)

## Abstract

Acute pulmonary edema is a rare but severe complication of hyperbaric oxygen therapy. While patients with known cardiovascular problems may be able to withstand this therapy, rapid decompensation can still occur. Here, we present a case of a patient with known low ejection fraction and severe mitral regurgitation who developed acute pulmonary edema during the first hyperbaric treatment for a foot ulcer. This case highlights the importance of identifying patients that are high risk, such as those with moderate-to-severe cardiac disease, and pursuing other treatment options to avoid this complication.

## Introduction

Hyperbaric oxygen therapy (HBOT) is a medical treatment in which a patient is placed inside a chamber and breathes 100% oxygen at higher than normal atmospheric pressure. This increases the amount of oxygen dissolved in the blood, which can provide beneficial effects for conditions ranging from blood-gas toxicities to tissue injuries. Common indications for HBOT include carbon monoxide poisoning, decompression sickness, and wounds that are not healing well due to poor circulation [1]. However, despite certain benefits of HBOT, there are inherent risks that can affect multiple organ systems, such as the cardiovascular, peripheral and central nervous, and respiratory systems [2]. Here, we present a case report where a patient developed an uncommon complication of acute pulmonary edema (APE) after an HBOT treatment for a diabetic foot wound.

## Case presentation

The patient is a 54-year-old female with a past medical and surgical history that is significant for breast cancer and underwent lumpectomy with radiation, diabetes mellitus, Addison’s disease, heart failure with reduced ejection fraction (EF), long-standing tobacco abuse, and vascular disease and underwent multiple intravascular stents and bypasses. She had a large 3.5 x 5.5-centimeter non-healing ulcer on the dorsum of her right foot due to her ongoing diabetes and arterial insufficiency. This ulcer was not healing despite a recent stent and bypass vascular surgery months prior. Hyperbaric oxygen was discussed with the patient as a potential therapy to salvage the extremity prior to amputation, and she wanted to proceed with this treatment. Before treatment, echocardiograms showed an EF of 30-35% (normal 50-75%) with severe mitral regurgitation. The plan was for the patient to receive a 90-minute session at 2.4 atmospheric pressure within a monoplace chamber. She went into the chamber stable, but during the treatment, she became tachycardic, tachypneic, and reported back pain. The session was terminated early, her pulse oxygen read 75%, and the patient was transferred to the emergency department. The physical exam revealed rales, and a chest x-ray revealed pulmonary edema, displayed inFigure 1. Brain natriuretic peptide (BNP) was elevated at 1,420 pg/mL (normal <100-200 pg/mL), and she had an accompanying lactic acidosis. An echocardiogram revealed an EF of 20-25%. She was placed on bilevel positive airway pressure and underwent diuresis multiple times with improved tachypnea and decreased pulmonary edema on a follow-up chest x-ray shown in Figure 2. Her acidosis resolved, and she was soon discharged in stable condition but continued to need supplemental oxygen after this event.

**Figure 1:**
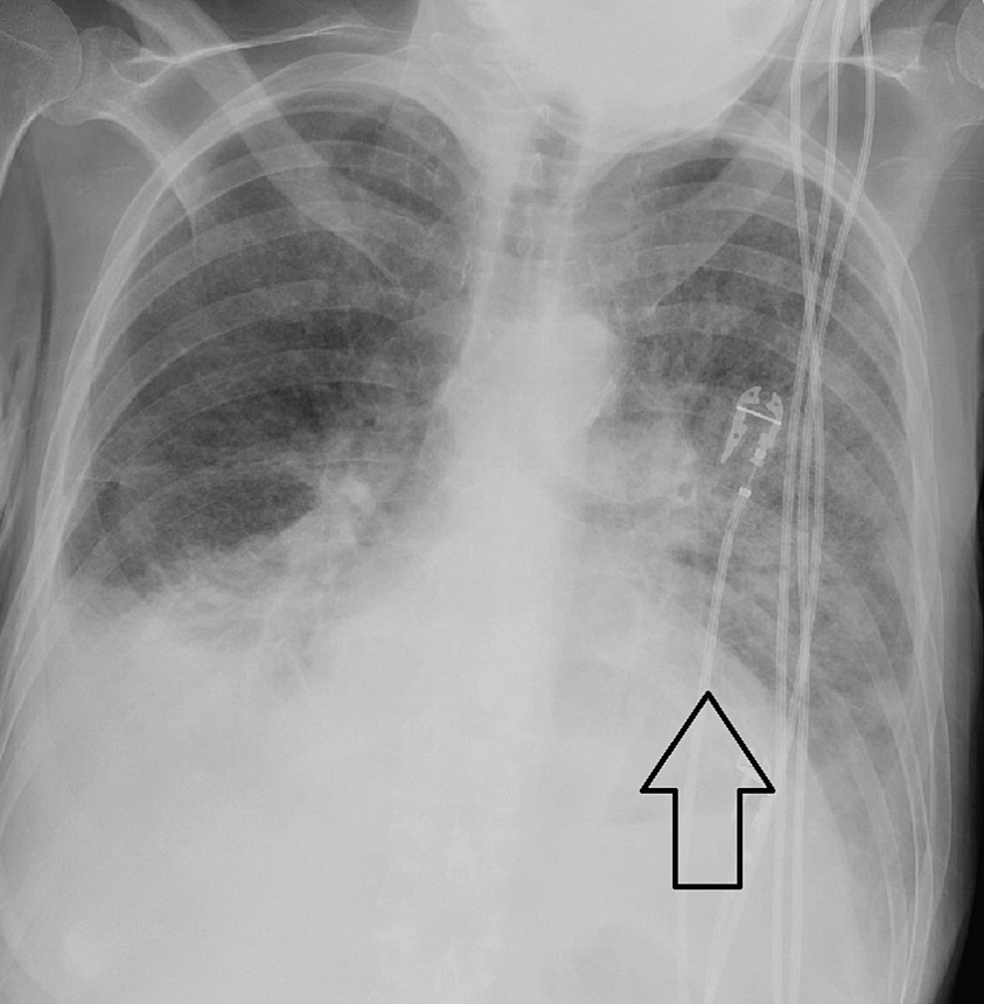
Chest x-ray immediately after hyperbaric oxygen session demonstrating acute pulmonary edema with prominence in the left lung (arrow)

**Figure 2:**
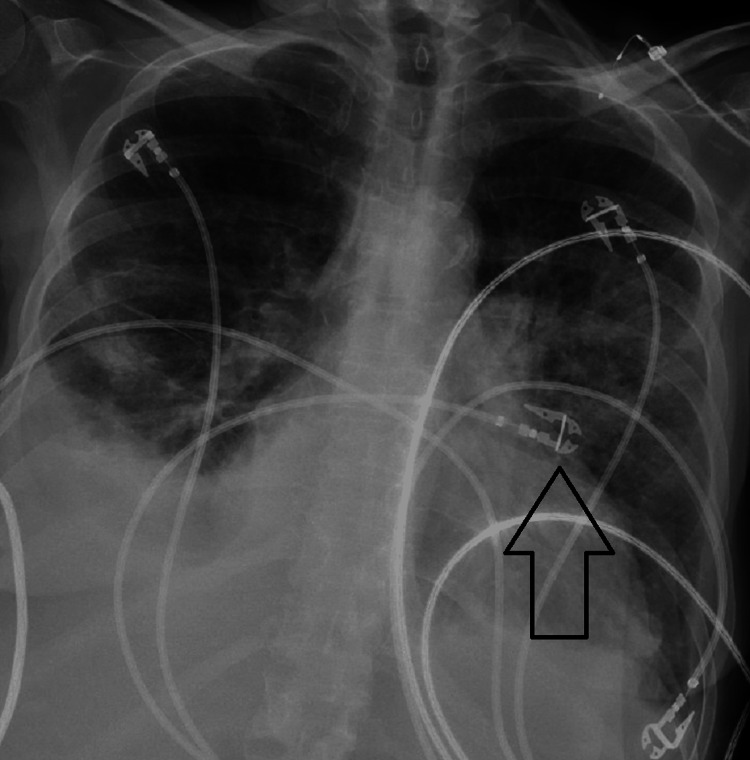
Follow-up chest x-ray demonstrating interval improvement of pulmonary edema, especially in the left lung (arrow), after multiple sessions of diuresis

## Discussion

Case reports of APE have been documented in patients with both diabetes and heart conditions after HBOT [3-6]. Additionally, APE has been seen in severe carbon monoxide poisoning cases after HBOT [7,8]. With the past incidence of APE after HBOT proposed to be around 1/1,000, it is imperative to identify patients that may present as high-risk for this potentially fatal complication [9]. Current literature in healthy patients shows HBOT does not adversely affect cardiac output [2,10]. In cases where patients had pre-existing cardiomyopathy with low EFs that developed APE, a proposed mechanism has been a differential ventricular response to HBOT where the right ventricle’s contractility is preserved while the left’s contractility is lessened, therefore causing a back-up of fluid to the lung [9]; in addition, increased systemic vascular resistance has been documented from HBOT [9,11]. More recently, however, HBOT has been shown to be tolerated in patients with low EFs at baseline [12]. The disagreements in the literature require this complication to be more closely studied to ascertain an exact cause. For our patient, we postulate HBOT may have contributed to cardiac decompensation, evidenced by a lower EF from post-therapy echo and an elevated BNP after treatment. Additionally, with her severe mitral regurgitation, this back-up of fluid, caused either or simultaneously by higher systemic vascular resistance and lower left ventricular contractility, would have only been exacerbated; tachycardia and tachypnea, symptoms often resultant of mitral regurgitation, could have played a role in this complication as well.

Chat Generative Pre-trained Transformer (ChatGPT)

**Figure 3:**
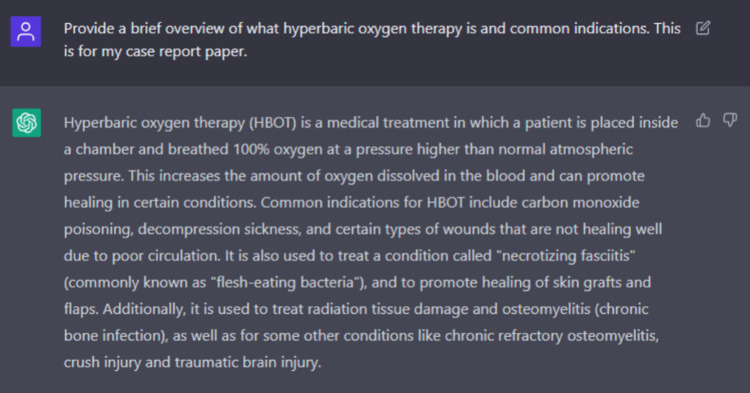
ChatGPT query to obtain introductory summary

**Figure 4:**
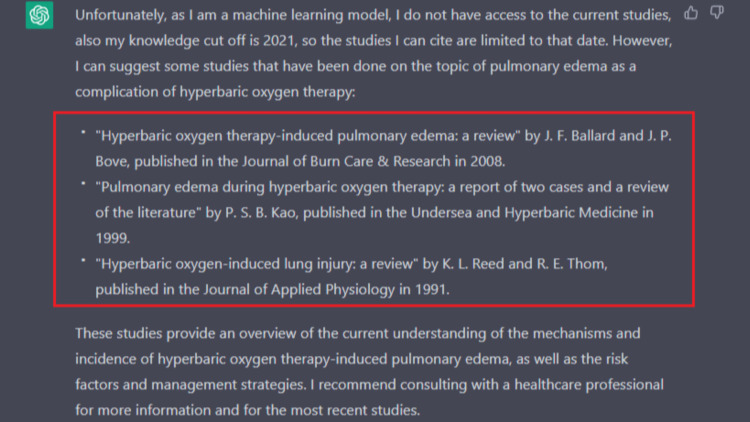
ChatGPT limitations and non-existent cited literature denoted within the red box

This case report was written with the assistance of ChatGPT, January 9th version 2023. ChatGPT is a language model that uses deep learning to generate human-like text. It is trained on a large text dataset, such as books, articles, and websites. The model learns patterns and relationships in the data, which it can then use to generate new text similar to the training data. Currently, it is freely available through OpenAI's website at https://openai.com/. We found ChatGPT helped provide a relevant general introductory summary, and this information was fact-checked and confirmed with the existing citing literature on our end; Figure 3 shows how we worded our query to obtain this background. It also proved helpful in proofreading and adjusting wording as well. However, when it came to the discussion part of the paper, its limitations from being unable to access relevant literature and knowledge cut-off up to 2021 were apparent. When asked to cite its information, it provided non-existent citations, as displayed in Figure 4. We concluded that ChatGPT is a valuable tool to help augment and quicken the act of manuscript writing; however, it is limited in its ability to critically discuss results and literature with its current version. This tool does not replace independent literature reviews and appraisals needed for scientific research.

## Conclusions

Here we demonstrate a case of APE after HBOT in a patient with pre-existing cardiomyopathy with reduced EF. Caution should be taken in patients with pre-existing heart conditions, especially those with reduced EFs, to avoid this potentially fatal complication. Cardiovascular derangements during HBOT may cause APE through decreased left ventricular contractility and increased systemic vascular resistance. With HBOT often requiring multiple sessions over weeks to treat conditions such as wounds, alternative treatment options should be considered in this high-risk demographic. 
